# Urologist and radiologist contributions to variation in prostate cancer detection at fusion biopsy

**DOI:** 10.1111/bju.70308

**Published:** 2026-05-07

**Authors:** Daniel Triner, Kevin B. Ginsburg, Ana Moser, Corinne Labardee, Howard Korman, Jason Domina, Michael L. Cher, James Peabody, Alice Semerjian, Andrei Purysko, Arvin K. George, Tudor Borza, Andrew E. Krumm, the Michigan Urological Surgery Improvement Collaborative (MUSIC)

**Affiliations:** ^1^ Department of Urology Stanford University Stanford CA USA; ^2^ Department of Urology Wayne State University School of Medicine Detroit MI USA; ^3^ Department of Radiology Karmanos Cancer Institute, Wayne State University Detroit MI USA; ^4^ Department of Urology Henry Ford Health System Detroit MI USA; ^5^ Department of Urology Michigan Medicine Ann Arbor MI USA; ^6^ Department of Learning Health Sciences University of Michigan Ann Arbor MI USA; ^7^ School of Information University of Michigan Ann Arbor MI USA; ^8^ Comprehensive Urology Royal Oak MI USA; ^9^ Section of Abdominal Imaging Diagnostic Institute, Cleveland Clinic Cleveland OH USA; ^10^ Brady Urological Institute Johns Hopkins University Baltimore MD USA

**Keywords:** prostate MRI, prostate cancer, Prostate Imaging‐Reporting And Data System, prostate cancer detection, Fusion biopsy

## Abstract

**Objectives:**

To quantify the relative contribution of radiologists and urologists to variability in clinically significant prostate cancer (CSPC) detection across multiparametric magnetic resonance imaging (mpMRI)‐guided prostate fusion biopsies and to determine whether this differed across Prostate Imaging‐Reporting And Data System (PI‐RADS) categories.

**Subjects, Patients and Methods:**

We analysed biopsy‐naïve men within the Michigan Urological Surgery Improvement Collaborative (MUSIC) who underwent fusion biopsy between August 2017 and November 2021. The primary outcome was the proportion of variance in CSPC detection (Gleason score ≥3 + 4) at targeted regions of interest that was explained by individual urologists and radiologists. We used generalised linear mixed‐effects models to partition variance and estimate intraclass correlation coefficients (ICCs) for radiologist‐ and urologist‐level effects for PI‐RADS score 3–5 lesions. We calculated the median odds ratio (MOR) to quantify the expected difference in odds of CSPC detection when comparing a randomly selected higher‐ vs lower‐detecting provider from the same distribution.

**Results:**

Among 1544 men with 2045 targeted lesions, mpMRIs were interpreted by 115 radiologists and biopsies performed by 86 urologists. For PI‐RADS score 3 lesions, urologists (ICC = 0.15, MOR = 2.07) accounted for greater variance in CSPC detection than radiologists (ICC = 0.07, MOR = 1.61). For PI‐RADS score 4, both urologist (ICC = 0.05, MOR = 1.49) and radiologist (ICC = 0.07, MOR = 1.61) contributed modestly. For PI‐RADS score 5, radiologists (ICC = 0.17, MOR = 2.19) explained a larger proportion of variance than urologists (ICC = 0.01, MOR = 1.19), suggesting individual radiologists meaningfully impact CSPC detection of high‐risk lesions.

**Conclusions:**

For PI‐RADS score 3 lesions, the specific urologist performing the biopsy was a strong source of variation, while for PI‐RADS score 5 lesions, the radiologist had greater influence on CSPC detection rates. Optimising MRI acquisition and interpretation, ensuring accurate fusion registration, and improving biopsy accuracy are critical to improving diagnostic consistency.

**Abbreviations**
CSPC: clinically significant prostate cancer
ICC: intraclass correlation coefficient
IQR: interquartile range
MOR: median odds ratio
mpMRI: multiparametric MRI
MUSIC: Michigan Urological Surgery Improvement Collaborative
PI‐RADS: Prostate Imaging‐Reporting And Data System
US: ultrasound

## Introduction

It is well established that the use of prostate multiparametric MRI (mpMRI) with MRI‐guided fusion software improves the detection of clinically significant prostate cancer (CSPC), defined as Gleason score ≥3 + 4, and may decrease over‐detection of clinically insignificant prostate cancers [1, 2]. Adoption of mpMRI‐guided fusion biopsy in prostate cancer diagnosis and management algorithms has been rapid in multiple phases of prostate cancer care. Much of the evidence supporting the use of prostate fusion biopsy comes from tertiary medical centres with limited numbers of radiologists interpreting mpMRI images and urologists performing prostate fusion biopsies. Given available evidence, little is known about whether clinical trial data regarding prostate fusion biopsy outcomes from expert centres are indicative of outcomes across the scope of radiological and urological practices in the United States.

Prostate fusion biopsy is a complex, multi‐step process involving imaging‐related factors (acquisition, lesion interpretation), and procedural‐related factors (image–ultrasound [US] co‐registration, and targeted sampling), all of which introduce potential variability. Reliability of prostate fusion biopsy is fundamental because variation in outcomes may impact treatment and surveillance decision making. Prior work has shown inter‐reader variability in individual radiologists’ interpretation of prostate mpMRI can influence CSPC detection with CSPC rates of Prostate Imaging‐Reporting And Data System (PI‐RADS) score 5 lesions ranging from 40% to 80% [3, 4]. Additionally, individual urologists performing prostate fusion biopsies may impact the likelihood of detecting CSPC [5, 6]. However, the degree to which these imaging‐related and procedural‐related factors contribute to variation in CSPC detection with prostate fusion biopsies, or how these contributions may differ among PI‐RADS scores, has not been rigorously assessed across real‐world practices. In this context, we sought to examine the variation in detection of CSPC with mpMRI‐guided fusion prostate biopsies at the imaging and procedural‐levels among a large and diverse statewide cohort of radiological and urological practices. Identifying, understanding, and addressing variability in prostate fusion biopsy outcomes may provide quality improvement opportunities for optimising CSPC detection.

## Subjects, Patients and Methods

### Study Design and Sample

The Michigan Urological Surgery Improvement Collaborative (MUSIC) is a statewide quality improvement collaborative that comprises >95% of urology practices in the state of Michigan and encompasses academic, hospital‐employed, and private practices. This analysis was a retrospective review of a prospectively maintained database on prostate fusion biopsy outcomes. Patient demographic and clinical information including prostate mpMRI with corresponding fusion biopsy data were prospectively entered in a web‐based clinical registry by trained abstractors at each clinical site by review of the primary medical record. Annual audits are performed at participating practices as part of quality control.

We included biopsy‐naïve men who underwent prostate mpMRI with a corresponding fusion biopsy between August 2017 and November 2021. Men with a history of a previous negative biopsy, history of prostate cancer undergoing repeat biopsy (such as a confirmatory biopsy or surveillance biopsy on active surveillance), or previous treatment of prostate cancer undergoing biopsy (such as radiation or ablation) were excluded from the study.

### The MRI Protocol and Fusion Biopsy

The mpMRI examinations were performed according to standard practices at each institution using a protocol that included T2‐weighted imaging, diffusion‐weighted imaging, and dynamic contrast‐enhanced imaging. PI‐RADS version 2 was utilised for interpretation by radiologists throughout the MUSIC [7]. MRIs that were not reported via the PI‐RADS scoring system were excluded.

All biopsies included in the study were software‐based MRI/US fusion‐guided biopsies via transrectal or transperineal approach. Biopsy approach and fusion biopsy platform were at the discretion of the operating urologist. In general, two to four cores were sampled per target lesion followed by a systematic 12‐core biopsy.

### Analysis

The primary objective was to assess variability in CSPC detection at targeted fusion biopsy cores across contributing radiologists and urologists for PI‐RADS score 3, 4, and 5 lesions. CSPC was defined as Gleason score ≥3 + 4. Our primary analytical approach involved fitting a Bayesian generalised linear mixed‐effects model for each PI‐RADS rating and calculating separate intraclass correlation coefficients (ICCs) for radiologists and urologists. A Bayesian approach was utilised in order to visualise an ICC estimate across a range of values, that is, draws, from the posterior distribution. Within each model, urologists and radiologists were treated as random effects. Each urologist and radiologist, therefore, was assumed to be a part of a distribution that had a mean of zero and a variance based on the weighted average differences among individual urologists and radiologists from each distribution's respective mean. To address common concerns with Bayesian models, we used multiple seeds and explored multiple values for each prior. All Bayesian models were fit using the ‘brms’ package in R 4.2.1 (R Foundation for Statistical Computing, Vienna, Austria) [8].

In the context of the present study, an ICC can be defined as the proportion of variance in CSPC findings that is attributable to urologists or radiologists, respectively. To account for the fact that generalised linear regression models do not estimate an overall residual variance, we used a ‘latent variable’ approach where residual variation is held constant at $\pi^{2}/3$ when calculating the ICC for each grouping variable in the model [9]. A latent variable approach is appropriate when the dichotomised dependent variable is the result of a threshold applied to a latent variable, such as an overall Gleason score with a CSPC cut‐off applied at ≥3 + 4.

To further contextualise provider‐level variance, we calculated separate median odds ratios (MORs) for radiologists and urologists within each PI‐RADS‐specific model. A MOR represents the expected change in odds of CSPC detection when comparing a randomly selected provider associated with higher detection rates vs a provider associated with lower detection rates drawn from the same distribution. A MOR of 1.0 indicates no provider‐level variation, or that randomly reassigning a patient from one provider to another would have minimal impact on their odds of CSPC detection. Larger values indicate greater differences between providers and less interchangeability, on average.

Our primary analysis involved the following steps: First, we estimated separate random effects only models by PI‐RADS rating and then visualised the model‐estimated CSPC detection rates across urologists and radiologists. We then fit separate mixed‐effects models by PI‐RADS rating that included the following patient‐level fixed effects: DRE (coded as positive, negative, or unknown), log (PSA), prostate volume (per mL), family history of prostate cancer (coded as positive, negative, or unknown), and age (in years). All patient‐level fixed effects were mean centred. To account for within‐patient correlation arising from multiple regions of interest, we randomly sampled one region of interest per patient per model, ensuring the independence of observations for estimating fixed effects. We took this step to avoid inflated standard error estimates for the fixed effects due to repeated observations for patients with multiple regions of interest. For both the random‐effects‐only and mixed‐effects models, we calculated a latent variable ICC per PI‐RADS rating and the overall *R* ^2^ for each model. Full regression tables are included in the Supporting Information.

## Results

A total of 2045 MRI lesions in 1544 biopsy‐naïve men met the inclusion criteria in the MUSIC registry. The median patient age was 67 years, the median (interquartile range [IQR]) pre‐biopsy PSA was 6.0 (4.6–8.4) ng/mL, and 12% of men had a positive pre‐biopsy DRE (Table 1). The median (IQR) pre‐biopsy MRI‐based prostate volume was 45 (33–65) mL. A total of 462 (23%) lesions were PI‐RADS score 3, 1094 (53%) were PI‐RADS score 4, and 489 (24%) were PI‐RADS score 5. A total of 115 radiologists performed mpMRI interpretations with a median (IQR) mpMRI reads of 7 (2–16) reads. We identified 86 urologists who performed prostate fusion biopsies. The median (IQR) number of fusion biopsies performed per urologist was 11 (2–23.75).

**Table 1:** Clinicopathological patient demographics.

Variable	Value	Missing, *n*
Age, years, median (IQR)	67 (61–72)	
Body mass index, kg/m^2^, median (IQR)	28.4 (25.8–31.9)	10
Prostate gland volume, mL, median (IQR)	45 (33–65)	44
PSA level, ng/mL, median (IQR)	6.0 (4.6–8.4)	16
Race/ethnicity, *n* (%)		
African American	98 (6.8)	
Caucasian	1289 (90.0)	
Other	50 (3.2)	
Total	1437	107
DRE, *n* (%)		
Positive	188 (12)	
Negative	1356 (88)	
Total	1544	
Family history, *n* (%)		
Yes	402 (24)	
No	1142 (76)	
Total	1544	
PI‐RADS score lesions, *n* (%)		
3	462 (23)	
4	1094 (53)	
5	489 (24)	
Total	2045	
Targeted biopsy pathology, per lesion, *n* (%)		
Gleason score ≥3 + 4	780 (38)	
Gleason score <3 + 4	1265 (62)	
Total	2045	

Urologists = 86; radiologists = 115; number of patients = 1544; imaging and biopsy pairs = 2045; race/ethnicity ‘Other’ = Asian, Native American, Native Hawaiian/Pacific Islander.

**Fig. 1:**
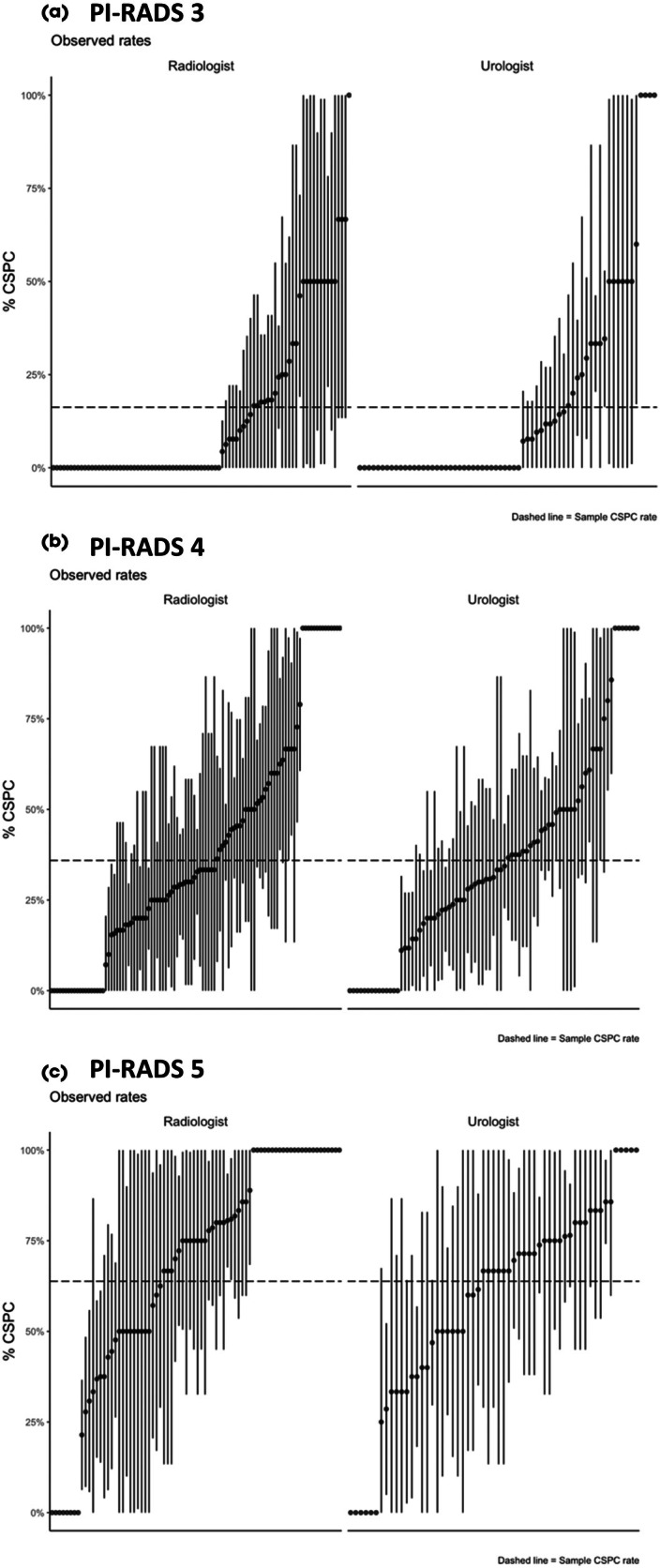
Observed detection of CSPC by radiologist and urologist for PI‐RADS score 3 **(a)**, score 4 **(b)**, and score 5 **(c)** lesions. The *x*‐axis corresponds to individual radiologists and urologists. The dashed line represents the cohort mean detection of CSPC and error bars represent the 95% CI.

The detection rate of CSPC on a per lesion basis was 16.2%, 35.9%, and 63.8% for PI‐RADS score 3, 4, and 5 lesions, respectively. Among radiologists, including low‐volume providers, there was wide variation in unadjusted CSPC detection at targeted fusion biopsy of PI‐RADS score 3 (0–66.7%), PI‐RADS score 4 (0–100%), and PI‐RADS score 5 (0–100%) lesions (Fig. 1a–c). Similarly, at the urologist level, the unadjusted CSPC detection rate of targeted biopsies ranged from 0% to 100% for PI‐RADS score 3, 0–100% for PI‐RADS score 4, and 0–100% for PI‐RADS score 5 (Fig. 1a–c).

For PI‐RADS score 3 lesions, urologists (ICC = 0.15) accounted for more variance in CSPC findings than radiologists (ICC = 0.07) and had higher MOR (urologist = 2.07, radiologist = 1.61). To contextualise the estimated ICC for urologists in relation to PI‐RADS score 3 lesions, an individual urologist who is 1 SD above the model‐based mean would be predicted to have a 22.6% chance of detecting CSPC, and a urologist 1 SD below the average urologist would be predicted to have a 5.6% chance of detecting CSPC with a fusion biopsy. Radiologists with an estimated ICC of 0.07, by contrast, showed less of a spread for radiologists estimated at 1 SD above (17.8%) or below (7.4%) the mean. For PI‐RADS score 4 lesions, urologists (ICC = 0.05) and radiologists (ICC = 0.07) explained similar, smaller amounts of variance and had similar, moderate MOR estimates (urologist = 1.49, radiologist = 1.61). For PI‐RADS score 5 lesions, radiologists had an ICC of 0.17 and MOR of 2.19, and urologists had an ICC of 0.01 and a MOR of 1.19. A radiologist 1 SD above the model‐based mean would have an 82.8% chance of being associated with a CSPC finding, and a radiologist 1 SD below would have a 47.8% chance.

The overall variance explained, *R* ^2^, by the mixed‐effects models for PI‐RADS score 3 was 8.8% conditional on patient‐level fixed effects alone and 19.2% when urologist and radiologist were included. For PI‐RADS score 4, 9.7% of the variance in CSPC findings were explained by patient fixed effects and 16.6% when urologist and radiologist were included. And for PI‐RADS score 5, 12.0% of the variance was explained by patient‐level factors and 20.3% when combined with urologist and radiologists.

**Fig. 2:**
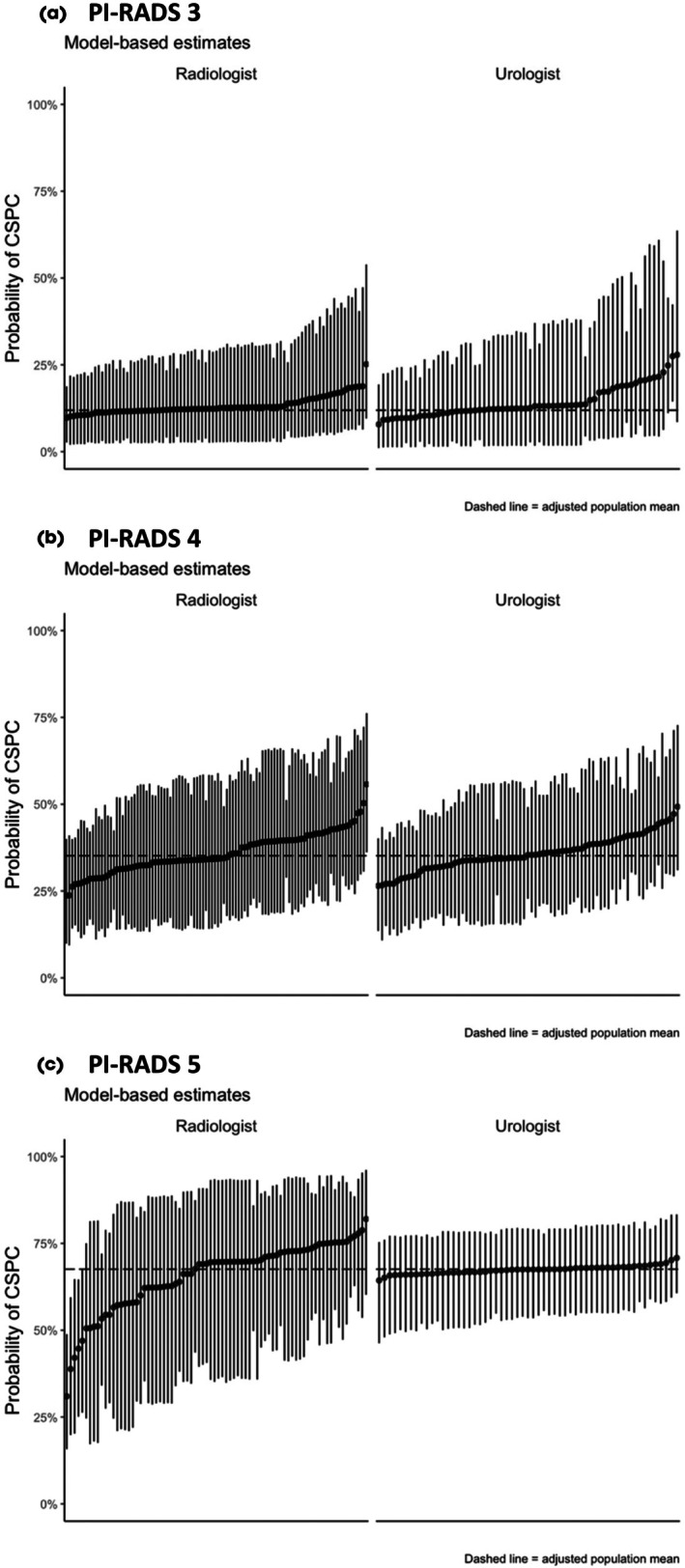
Model‐based CSPC rates by radiologist and urologist accounting for the influence of individual radiologists and urologists. The *x*‐axis corresponds to individual radiologists and urologists. The dashed line represents the predicted probability of CSPC among the cohort. Error bars display the 95% credible intervals.

Figure 2 shows the model‐adjusted probability of CSPC for each urologist and radiologist. The dashed line represents the estimated average CSPC rate. While there is notable variability in the unadjusted CSPC rates across PI‐RADS scores (Fig. 1a–c), Fig. 2 considers the overall CSPC rate for a given PI‐RADS lesion within the sample and adjusts each urologist's and radiologist's associated CSPC rate based on the number of observations associated with them. Radiologists or urologists whose 95% credible intervals do not touch the estimated population mean in Fig. 2 can be interpreted as having meaningfully different associations with CSPC findings than the estimated mean. There was one urologist associated with PI‐RADS score 3 lesions who could be identified as different from the sample, one radiologist for PI‐RADS score 4 lesions, and four radiologists for PI‐RADS score 5 lesions who could all be characterised as distinct from each sample's respective mean CSPC rate.

## Discussion

Reliability of prostate fusion biopsy is critical as this can impact decision‐making (treatment vs surveillance) and intensity of treatment administered. While several studies have evaluated the role and integration of fusion biopsy in the prostate cancer diagnostic algorithm, less is known about how imaging‐ and procedural‐level factors influence variability in biopsy outcomes across diverse clinical environments. This is the largest analysis to quantify the contribution of radiologists and urologists to CSPC detection at prostate fusion biopsy [3, 5]. Our findings demonstrate wide variation in detection of CSPC irrespective of PI‐RADS score and this variation in CSPC detection during prostate fusion biopsy arises from both imaging (radiologist)‐ and procedural (urologist)‐related domains. While urologists explained more variation in CSPC detection for PI‐RADS score 3 lesions, radiologists explained more variation for PI‐RADS score 5 lesions.

To understand the magnitude of the ICC value in this context, for PI‐RADS score 3 lesions, there was a 17% difference between urologists estimated at 1 SD above and below the average urologist. For PI‐RADS score 5 lesions, the difference between a radiologist 1 SD above the mean (82.8% CSPC rate) vs 1 SD below the mean (47.8% CSPC rate) was 35%. While there were notable ranges between urologists and radiologists for specific PI‐RADS findings descriptively and based on statistical modelling, urologists and radiologists accounted for a modest proportion of the total variation in CSPC detection for the per‐lesion analysis using random‐effects only (Urologist = 15% for PI‐RADS score 3 and radiologist = 17% for PI‐RADS score 5), and neither explained a meaningful proportion of variation for PI‐RADS score 4 lesions. Results from the mixed‐effects models that included patient‐level factors showed that 9% of the overall variance was explained by patient‐level factors and when the specific urologist or radiologist was accounted for, 19.2% of the overall variance was explained. Similar overall proportions were observed for PI‐RADS score 4 and 5 ratings. Thus, even fully specified models explained only ~20% of total variance in CSPC detection, highlighting that substantial variability arises from sources not captured in our models.

A key takeaway from these results is that diagnosing CSPC is dependent upon imaging‐ and procedural‐related factors. Current nomograms that leverage PI‐RADS ratings do not account for a potential influence of urologists and radiologists in the detection of CSPC. While the absolute variance explained by individual radiologists and urologists may appear minor, our current understanding of prostate fusion biopsy outcomes does not assume any contribution from individual practitioners. Prior studies in prostate MRI have shown that even moderate inter‐reader variation can meaningfully affect outcomes [10]. We therefore consider an ICC of 0.15 and MOR of 2.07 for urologists and PI‐RADS score 3 ratings as well as an ICC of 0.17 and MOR of 2.19 for radiologists and PI‐RADS score 5 ratings a notable finding, suggesting providers are not interchangeable for these ratings.

Prior work by Sonn et al. [3] has shown that there is substantial radiologist‐level variability in mpMRI interpretation, PI‐RADS score assignment, and CSPC detection across a small number of radiologists at a single institution. Another large and multicentre study found a high degree of radiologist variability in positive predictive value of lesions with PI‐RADS scores ≥3 [4]. Radiologist experience and specialisation may contribute to the variability in PI‐RADS interpretation [11, 12, 13]. At the urologist‐level, a single‐institution study showed similar CSPC detection rates among five individual urologists. However, when performance was evaluated at the PI‐RADS lesion‐level there was a significant difference in CSPC detection for PI‐RADS score 4 lesions [6]. At the urologist level, experience and expertise with fusion biopsy is predictive of CSPC detection [14]. Patel et al. [5] evaluated urologists’ and radiologists’ contribution to fusion‐biopsy CSPC at a single centre (10 radiologists and five urologists) and found wide variation in CSPC detection rates with radiologists contributing more to the variation in outcomes relative to urologists across all PI‐RADS lesions. We expanded on this prior work to quantify the degree of variation at specific PI‐RADS lesions. We found wide variability in CSPC detection with urologists contributing more to variation in PI‐RADS score 3 lesions and little to PI‐RADS score 5 lesions and radiologists contributing substantially to variation to CSPC detection in PI‐RADS score 5 lesions.

Our study has several quality improvement and clinical implications. For example, consider the unadjusted CSPC detection rate for PI‐RADS score 5 lesions for urologists, which spans from 0% to 100%. Without understanding the contribution of how radiologists influence these outcomes, several urologists could be criticised for poor technical skills and ability to adequately sample the region of interest. Yet, the model‐adjusted detection rate for urologists for PI‐RADS score 5 lesions show minimal urologist‐level variation (ICC = 0.01, MOR = 1.19), which suggests that urologists have limited influence on downstream detection rates. Clinically, this is rather intuitive as PI‐RADS score 5 lesions are generally large and sampling them is technically less challenging; the radiologist who is reading and calling the lesion a PI‐RADS score 5 contributes notably to the detection of CSPC among men with PI‐RADS score 5 lesions. An MOR of 2.19 indicates that radiologists interpreting PI‐RADS score 5 lesions are not interchangeable, i.e., there are potentially key radiologist‐to‐radiologist differences. Among radiologists, true outliers can be detected, that is, there are radiologists whose individual average CSPC rate is below the sample's average (Fig. 2), indicating that they could be ‘over‐calling’ PI‐RADS score 5 lesions.

Similarly for PI‐RADS score 3 lesions, multiple radiologists and urologists have a CSPC detection rate much higher than expected in unadjusted analysis; however, these providers perform much closer to the mean and expected rate when adjusting for patient‐ and provider‐level effects. Urologists contribute more to the variation in the detection of CSPC in patients with PI‐RADS score 3 lesions and a MOR of 2.07 demonstrates non‐ignorable urologist‐by‐urologist variation, which suggests that educational efforts to aid in technical targeted biopsy skills could be directed in this disease space. Clinically, PI‐RADS score 3 lesions are smaller and as a result, the technical ability to accurately co‐register and target the lesion by the urologist may explain the procedural contribution to the detection of CSPC in these lesions. Lastly, we believe these data show it is imperative to analyse the system as a whole, rather than an individual in isolation to identify areas for improvement. As discussed above, variation in prostate fusion biopsy outcomes is spread across patients, urologists, and radiologists, which highlights the complexity in studying prostate fusion biopsy outcomes. Establishment and maintenance of a multidisciplinary programme to collect and review urologist and radiologist outcomes may decrease variation in CSPC detection. Knowing one's own institutional data and outcomes is essential information during patient counselling and medical decision‐making regarding prostate mpMRI [15].

Our study has several limitations. First, biopsies were taken by urologists of various levels of skill and training; there was not a standardised fusion biopsy method or platform utilised across the MUSIC. Similarly, there was no standardised method for radiology review and reporting of mpMRI images, although all radiologists did use the PI‐RADS version 2 system. In addition, multiple MRI scanners at multiple institutions contributed imaging for this study without standardisation of MRI acquisition or image processing. Moreover, the MUSIC does not collect data on radiologist or urologist demographics including prior training and years in practice. However, the data presented here are representative of how MRI and fusion biopsy are being used in real‐world practice. While our analysis focused exclusively on CSPC detection in targeted cores to isolate the performance of MRI‐guided sampling, systematic biopsies can identify additional CSPCs from areas adjacent to or remote from MRI‐visible lesions [16]. Our results may underestimate overall CSPC detection and should be interpreted as reflecting variability in targeting rather than total biopsy yield. Additionally, prior work has shown that roughly 20% of CSPC cases detected by systematic biopsy alone are located immediately adjacent to or on the same side as the targeted lesion. These ‘misses’ likely represent the combined influence of multiple factors—including lesion characterisation by the radiologist, targeting accuracy by the urologist, and intrinsic limitations of fusion biopsy platforms—underscoring that detection variability is a shared rather than single‐actor phenomenon.

Lastly, the present study did not include men with negative MRI examinations. While the present analysis focused on MRI‐visible lesions to isolate operator and reader effects, negative MRI cases are clinically important given that a subset of men with negative imaging still harbour CSPC. Ongoing work within the MUSIC aims to quantify variability in CSPC detection among patients with negative MRI to provide a more comprehensive assessment of MRI performance in real‐world practice. Nonetheless, the strength of the analysis is the reflection of real‐world clinical practice and understanding how MRIs and targeted biopsies are being performed clinically.

Despite these limitations, our results emphasise that imaging‐ and procedural‐related factors jointly shape fusion biopsy outcomes. For patients with PI‐RADS score 5 lesions, radiologists contributed most to the variation in outcomes, while the urologists performing fusion biopsies contributed negligibly. For PI‐RADS score 4 lesions, radiologists and urologists contributed equally, and for PI‐RADS score 3 lesions, urologists contributed most to the variation in the detection of CSPC. While it is essential to understand urologists’ and radiologists’ contribution to variation in fusion biopsy outcomes, a large proportion of variation in these outcomes can be attributed to other sources and should be explored further. Moreover, interdisciplinary collaboration between radiologists, urologists, and pathologists is essential for comprehensive lesion assessment, integrating imaging findings with clinical data, and histopathological analysis to optimise patient management strategies.

## Disclosure of Interests

None declared.

## Funding

Support for the MUSIC is provided by BCBSM as part of the BCBSM Value Partnerships programme.

## Supporting information


**Table S1.** The PI‐RADS score 3 random‐effects model.
**Table S2.** The PI‐RADS score 4 random‐effects model.
**Table S3.** The PI‐RADS score 5 random‐effects model.
**Table S4.** The PI‐RADS score 3 patient fixed‐effects model.
**Table S5.** The PI‐RADS score 4 patient fixed‐effects model.
**Table S6.** The PI‐RADS score 5 patient fixed‐effects model.
