# Supplementary material for: Urologist and radiologist contributions to variation in prostate cancer detection at fusion biopsy
Source: BJU Int. 2026 May 7;138(1):156–63. doi: 10.1111/bju.70308 (PMC13244926; doi:10.1111/bju.70308)
Supplement: Supplementary file 1 — Table S1. The PI‐RADS score 3 random‐effects model. Table S2. The PI‐RADS score 4 random‐effects model. Table S3. The PI‐RADS score 5 random‐effects model. Table S4. The PI‐RADS score 3 patient fixed‐effects model. Table S5. The PI‐RADS score 4 patient fixed‐effects model. Table S6. The PI‐RADS score 5 patient fixed‐effects model. [file BJU-138-156-s001.docx]

**Supplementary Methods**

**Inclusion criteria**

There were initially 2,473 multiparametric magnetic resonance imaging (mpMRI)-guided prostate fusion biopsy pairs identified between August 2017 and November 2021. Across these images, there were initially 1,544 patients identified. These dates represented when the Michigan Urologic Improvement Collaboratives (MUSIC) began retaining negative biopsy results, and November 2021 represented a quality assurance task related to understanding radiologists who performed imaging within MUSIC.

**Random effects only models**

Tables S1–S3 show the model results for the random effects only models across by PI-RADS rating.

**Table S1.** PIRADs 3 random effects model

|  | **Estimate** | **Low 95% CI** | **High 95% CI** |
| --- | --- | --- | --- |
| Intercept | -2.03 | -2.60 | -1.58 |
| Urologist (Random Effect) | 0.80 | 0.25 | 1.46 |
| ICC | 0.15 |  |  |
| Radiologist (Random Effect) | 0.55 | 0.04 | 1.12 |
| ICC | 0.07 |  |  |
| R^2^ | 0.10 | 0.03 | 0.19 |

**Table S2.** PIRADs 4 random effects model

|  | **Estimate** | **Low 95% CI** | **High 95% CI** |
| --- | --- | --- | --- |
| Intercept | -0.61 | -0.85 | -0.38 |
| Urologist (Random Effect) | 0.45 | 0.11 | 0.74 |
| ICC | 0.05 |  |  |
| Radiologist (Random Effect) | 0.52 | 0.26 | 0.79 |
| ICC | 0.07 |  |  |
| R^2^ | 0.08 | 0.04 | 0.12 |

**Table S3.** PIRADs 5 random effects model

|  | **Estimate** | **Low 95% CI** | **High 95% CI** |
| --- | --- | --- | --- |
| Intercept | 0.74 | 0.42 | 1.09 |
| Urologist (Random Effect) | 0.21 | 0.01 | 0.55 |
| ICC | 0.01 |  |  |
| Radiologist (Random Effect) | 0.83 | 0.52 | 1.21 |
| ICC | 0.17 |  |  |
| R^2^ | 0.15 | 0.08 | 0.21 |

**Mixed effect models analyses**

Mixed effects models adjusted for patient factors associated with clinically significant prostate cancer (CSPC) biopsy findings. For these models, the intra-class correlation (ICC) can be interpreted as a conditional ICC that characterizes the residual variation not explained by the fixed effects. Whereas an unconditional ICC can be understood as the degree to which CSPC findings are correlated within a given urologist or radiologist for a specific PI-RADS rating, a conditional ICC can be understood as the correlation of CSPC findings within a urologist or radiologist when patient factors are held constant. Tables S4–S6 show the regression results for these models.

**Table S4.** PIRADs 3 patient fixed effects model

|  | **Estimate** | **Low 95% CI** | **High 95% CI** |
| --- | --- | --- | --- |
| Intercept | -1.92 | -2.50 | -1.46 |
| DRE | 0.68 | -0.28 | 1.61 |
| PSA (log) | 0.85 | 0.32 | 1.40 |
| Gland Vol. | -1.82 | -2.52 | -1.15 |
| Fam hist. | 0.26 | -0.39 | 0.90 |
| Age | 0.06 | 0.02 | 0.10 |
| Urologist (Random Effect) | 0.69 | 0.08 | 1.45 |
| ICC | 0.12 |  |  |
| Radiologist (Random Effect) | 0.53 | 0.03 | 1.19 |
| ICC | 0.07 |  |  |
| Conditional on random effects R^2^ | 0.19 | 0.11 | 0.28 |
| Marginal of random effects R^2^ | 0.09 | 0.03 | 0.15 |

**Table S5.** PIRADs 4 patient fixed effects model

|  | **Estimate** | **Low 95% CI** | **High 95% CI** |
| --- | --- | --- | --- |
| Intercept | -0.60 | -0.87 | -0.34 |
| DRE | 0.42 | -0.06 | 0.91 |
| PSA (log) | 0.65 | 0.34 | 0.97 |
| Gland Vol. | -1.28 | -1.65 | -0.92 |
| Fam hist. | 0.02 | -0.33 | 0.37 |
| Age | 0.04 | 0.01 | 0.06 |
| Urologist (Random Effect) | 0.44 | 0.07 | 0.79 |
| ICC | 0.05 |  |  |
| Radiologist (Random Effect) | 0.56 | 0.17 | 0.91 |
| ICC | 0.08 |  |  |
| Conditional on random effects R^2^ | 0.17 | 0.12 | 0.21 |
| Marginal of random effects R^2^ | 0.10 | 0.06 | 0.13 |

**Table S6.** PIRADs 5 patient fixed effects model

|  | **Estimate** | **Low 95% CI** | **High 95% CI** |
| --- | --- | --- | --- |
| Intercept | 0.43 | 0.10 | 0.77 |
| DRE | 0.51 | -0.07 | 1.10 |
| PSA (log) | 0.98 | 0.57 | 1.40 |
| Gland Vol. | -0.71 | -1.21 | -0.22 |
| Fam hist. | -0.09 | -0.61 | 0.43 |
| Age | 0.06 | 0.03 | 0.09 |
| Urologist (Random Effect) | 0.20 | 0.01 | 0.56 |
| ICC | 0.01 |  |  |
| Radiologist (Random Effect) | 0.69 | 0.30 | 1.10 |
| ICC | 0.13 |  |  |
| Conditional on random effects R^2^ | 0.20 | 0.14 | 0.27 |
| Marginal of random effects R^2^ | 0.12 | 0.07 | 0.17 |
